# The Crucial Role of DNA Methylation and MeCP2 in Neuronal Function

**DOI:** 10.3390/genes8050141

**Published:** 2017-05-13

**Authors:** Maria Fasolino, Zhaolan Zhou

**Affiliations:** Department of Genetics, Perelman School of Medicine at the University of Pennsylvania, Philadelphia, PA 19104, USA; fasolino@mail.med.upenn.edu

**Keywords:** DNA methylation, 5-methylcytosine (5mC), 5-hydroxymethylcytosine (5hmC), methyl-CpG-binding protein 2 (MeCP2), Rett Syndrome (RTT), DNA methyltransferase (DNMT), ten-eleven translocation (TET) methylcytosine dioxygenase, neuroplasticity, long-term potentiation (LTP)

## Abstract

A neuron is unique in its ability to dynamically modify its transcriptional output in response to synaptic activity while maintaining a core gene expression program that preserves cellular identity throughout a lifetime that is longer than almost every other cell type in the body. A contributing factor to the immense adaptability of a neuron is its unique epigenetic landscape that elicits locus-specific alterations in chromatin architecture, which in turn influences gene expression. One such epigenetic modification that is sensitive to changes in synaptic activity, as well as essential for maintaining cellular identity, is DNA methylation. The focus of this article is on the importance of DNA methylation in neuronal function, summarizing recent studies on critical players in the establishment of (the “writing”), the modification or erasure of (the “editing”), and the mediation of (the “reading”) DNA methylation in neurodevelopment and neuroplasticity. One “reader” of DNA methylation in particular, methyl-CpG-binding protein 2 (MeCP2), is highlighted, given its undisputed importance in neuronal function.

## 1. The Adaptability of Gene Expression Programs in Response to Neuronal Activity

Neuroplasticity is the ability of the brain to alter neuronal function in response to environmental input. This adaptability, expressed as long-lasting plasticity, is the basis of learning and memory formation. Two neural mechanisms that are essential for long-lasting plasticity are long-term depression (LTD) and long-term potentiation (LTP), which decrease and increase synaptic strength, respectively, in response to activity [1,2]. Here we will focus on LTP, as key molecules in this process are frequently referenced throughout this review. 

LTP is generally broken down into two phases: a transient “early” phase (30–60 min) that is dependent upon the activation of pre-existing proteins and a stable “late” phase (hours, days, or even weeks) that is contingent upon new gene transcription and translation [1]. The induction of early phase LTP is dependent upon postsynaptic depolarization that leads to an influx of calcium (Ca^2+^) through NMDA receptors (NMDARs). Ca^2+^ entry in turn triggers a biochemical cascade starting with the activation (via phosphorylation) of calcium/calmodulin-dependent protein kinase II (CaMKII), which translocates to active synapses, where it is thought to bind to NMDARs, leading to the activation (via phosphorylation) of AMPA receptors (AMPARs). CaMKII is also thought to play a role in the activation of the RAS-extracellular signal-regulated kinase (ERK) (RAS-ERK) pathway, which subsequently leads to an increase in the insertion of AMPARs into the synapse. Together, these CaMKII-dependent actions increase the conductance of Ca^2+^ through AMPARs in stimulated spines, which triggers a molecular cascade that strengthens potentiated spines [1,3]. An important modulator of the CaMKII cascade is protein phosphatase 1 (PP1), which at basal conditions inactivates CaMKII via dephosphorylation; however, in response to neuronal activity and subsequent LTP induction, cyclic adenosine monophosphate (cAMP) activates protein kinase A, which in turn phosphorylates inhibitor 1 (I-1) that inhibits PP1, leading to an increase in CaMKII phosphorylation and activity [1,3,4,5]. 

Similarly to the CaMKII cascade, many of the other early phase LTP signaling cascades converge upon the activation of ERK, which in turn activates various biochemical pathways that are essential for late phase LTP. One group of proteins activated by ERK are transcription factors, such as cAMP response element binding protein (CREB), FBJ osteosarcoma oncogene B (FOSB), and nuclear factor kappa-light-chain-enhancer of activated B cells (NF-κB), which subsequently induce gene expression changes essential for the potentiation of active synapses, such as immediate early response genes, ion channels, structural proteins, and neurotrophins [2,6]. One neurotrophin that is particularly important in the maintenance of LTP is brain-derived neurotrophic factor (BDNF), which acts through mitogen-activated protein kinase (MAPK)/ERK kinase (MEK) to increase the expression of activity regulated cytoskeletal-associated protein (*Arc*), a gene important in cytoskeleton organization at activated synapses [6,7]. It is during this late phase of LTP where DNA methylation is thought to contribute to the regulation of the expression of key genes necessary for the maintenance of long-term neuronal plasticity, and in this review, we will summarize the substantial body of work supporting this notion. We will begin with an overview of the important forms of DNA methylation in the mammalian brain.

## 2. Types of DNA Methylation in Neurons

### 2.1. 5mCG: A Repressive Epigenetic Mark

DNA methylation is an epigenetic mechanism that allows for sustained adaptability of gene expression in response to developmental or environmental factors; it plays an essential role in various biological functions, such as regulation of gene transcription and the establishment and maintenance of cellular identity [8,9,10]. DNA methylation at the 5-carbon of cytosine (5mC) is widely distributed throughout the mammalian genome, with ~5% of cytosines being methylated in the adult mouse brain. In mammals, methylation predominantly occurs at cytosine-phosphate-guanine dinucleotides (CpGs), with methylation occurring at 60–90% of CpGs depending on the tissue type, and in the mammalian brain, ~62% of CpGs are methylated (a methylated CpG will subsequently be referred to as 5mCG) [11,12,13,14] (Table 1). DNA methylation is deposited on cytosine by the family of enzymes known as the DNA methyltransferases (DNMTs). This enzyme family is divided into two broad classes: the *de novo* DNMTs, DNMT3A and DNMT3B, which establish methylation patterns on unmethylated DNA; and the maintenance DNMT, DNMT1, which preserves existing methylation patterns by methylating hemimethylated DNA [15]. Whereas *Dnmt3a* and *Dnmt1* are expressed in both the embryonic and adult stages of the brain, *Dnmt3b* is detectable only during early neurogenesis [16,17], suggesting that particular DNMTs play a role in neuronal function at specific times over neuronal development and maturation.

5mCG in all somatic cell types, including neurons, correlates with the repression of genes and repetitive DNA regions given whole genome bisulfite sequencing (WGBS) findings, which is a method used to determine genome-wide methylated cytosine sites, that 5mCG is enriched at intergenic regions, silenced genes, and repetitive DNA regions and is depleted at enhancers, promoters, and actively expressed gene bodies [11,13,18,19] (Table 1). Additionally, 5’-upstream, gene body, and 3’-downstream 5mCG levels inversely correlate with gene expression in neurons [20]. Furthermore, 5mCG patterns are neuronal type-specific, suggesting that this epigenetic mark regulates gene expression in a cell type-specific manner [13,20]. This epigenetic mark is also important in nuclear organization and compartmentalization given that the methylation of satellite repeats is an important step in the formation of heterochromatic regions [21,22].

### 2.2. An Introduction to Additional Neuronally Enriched Forms of DNA Methylation

Historically, DNA methylation was thought to be a stable, repressive covalent modification, existing predominantly, if not exclusively, as 5mCG. However, this view has dramatically changed over the past few years with the discovery that neurons, unlike other adult somatic cells, accumulate two other forms of DNA methylation over development, 5-hydroxymethylcystosine (5hmC) and non-CG methylation (5mCH, where H = A, T, or C). 5hmC levels in neurons are up to 10-fold higher than other cell types, reaching almost 20% of all DNA methylation in the mouse frontal cortex [23,24,25,26,27] (Table 1). Even though 5mCH is nearly absent in other cell types, except embryonic stem cells (ESCs), 5mCH accounts for up to 53% of all DNA methylation in adult human neurons, making it the predominant form of DNA methylation, and ~25–40% of all DNA methylation in adult mouse neurons (Table 1). Although the overall percentage of all CH dinucleotides that are methylated is relatively low (~1.3% of CH dinucleotides in adult mouse neurons) compared to those methylated in the CG context (~62% of CG dinucleotides in adult mouse neurons), the relative depletion of CG dinucleotides in eukaryotic genomes enables the high overall abundance of methylation in the CH context in neurons [11,12,13,14,20,28,29,30]. Therefore, 5hmC and 5mCH have added important new dimensions in understanding epigenetic regulation of neuronal function.

### 2.3. 5hmCG: A Neuronally Enriched Form of DNA Methylation Associated with Gene Activation

5mC is oxidized by the ten-eleven translocation (TET) methylcytosine dioxygenase family of enzymes to form 5hmC [24]. Three Tets are expressed in the brain (*Tet1–3*), with *Tet2* and *Tet3* mRNA levels being considerably higher than *Tet1* postnatally [15,31]. Recently developed sequencing approaches have enabled the genomic detection of 5hmC, providing invaluable insight into the biological function of this epigenetic mark. Approximately 0.9% of all cytosines are hydroxymethylated in the mouse brain (Table 1), predominantly (>98%) occurring in the CG context [13]. 5hmC is enriched at transcriptionally active sites, such as gene bodies, transcriptional end sites, DNase I hypersensitive sites (DHSs), and both active and poised enhancers, and is depleted at promoters and major satellite regions [13,32,33] (Table 1). Additionally, gene expression levels positively correlate with intragenic 5hmC levels [13,26,34]. These features suggest that 5hmC is involved in gene activation, which starkly contrasts with the biological function of 5mCG. The importance of 5hmC in neuronal maturation and function is highlighted by the fact that 5hmC levels increase nearly tenfold over development [13], and genes enriched with 5hmC in the mammalian brain relative to other tissues are related to synaptic function [35]. The substantial body of literature regarding the importance of the 5hmC and TETs in neuronal function will be reviewed below. 

### 2.4. 5mCH: A Repressive DNA Methylation Mark in Neurons

Whereas 5mCG is established by three DNMTs (DNMT1, DNMT3a, and DNMT3b) and levels remain unchanged during development [15], 5mCH is catalyzed primarily by DNMT3a [36] and levels increase during synaptogenesis in the brain, which coincides with an increase in *Dnmt3a* mRNA levels during this same time period [13,29]. Further supporting the unique role of *Dnmt3a* in 5mCH formation is a study in which exogenous *Dnmt3a* expression in *Drosophila melanogaster* led to the formation of *de novo* 5mCH [36]. Additionally, conditional deletion of *Dnmt3a* from the brain during early development results in a significant reduction in 5mC, specifically in the 5mCA and 5mCT contexts, but not in the 5mCG context [29,37]. Similarly to 5mCG, 5’-upstream, gene body, and 3’-downstream 5mCH levels inversely correlate with gene expression, with gene body 5mCH outperforming 5mCG as an indicator of transcriptional levels in the brain [20,29] (Table 1). Additionally, 5mCH patterns are cell type-specific, which is similar to 5mCG, but 5mCH is an even better signal than 5mCG in this regard [20,29]. 5mCH-mediated repression has been confirmed with the use of a methylated quantitative reporter assay in hippocampal neurons [29]. Furthermore, 5mCH is abundant at intergenic regions and repetitive DNA regions, whereas it is depleted from sites bound by transcription factors [20,29,38] (Table 1). Taken together, this suggests that the brain-specific establishment and mediation of this epigenetic mark may contribute to a unique regulatory effect in neurons [30], as will be discussed below.

## 3. The Necessity of DNA Methylation in Neurodevelopment

The importance of DNA methylation in neurodevelopment has been demonstrated by studies in which the loss of key enzymes that regulate DNA methylation, DNMTs and TETs, leads to deficits in neuronal function. Embryonic deletion of *Dnmt1* from forebrain neural progenitors in mice results in hypomethylation, dysregulation of gene expression, deficits in cerebral cortical formation and maturation, increased dendritic branching, reductions in LTP, defects in learning and memory, and severe embryonic and early postnatal neurodegeneration [8,10]. Similarly, embryonic deletion of *Dnmt3a* from the entire central nervous system (CNS) of mice also results in neuronal dysfunction, such as hypoactivity, motor abnormalities, decreased grip strength, a reduction in motor neuron number, and a shortened lifespan [39]. Human genetics also supports the importance of DNMTs in neuronal function as mutations in *DNMT1* have been linked to hereditary sensory and neuropathy with dementia and hearing loss [40], *DNMT3A* with an overgrowth syndrome with intellectual disability [41], and *DNMT3B* with immunodeficiency, centromere instability, and facial anomalies (ICF) syndrome, in which a large percentage of patients have intellectual disability [42,43,44].

Similarly to DNMTs, various studies have also demonstrated the importance of TETs in brain development. *Tet1* constitutive knockout mice or mice lacking the catalytic dioxygenase domain of *Tet1* display memory deficits, a reduction in the expression of genes associated with neurogenesis and neuronal activity (along with hypermethylation at the promoters of this gene group), LTP defects, increased LTD, and a reduction in the proliferating potential of neural progenitor cells (NPCs) [45,46]. The two other TET family members, TET2 and TET3, also influence neuronal development, as the knockdown of *Tet2* and *Tet3* via electroporation of short hairpin RNAs (shRNAs) into the cortex of mice leads to defects in the progression of differentiated neurons from the subventricular zone [47]. 

Given that these are embryonic perturbations of DNMTs and TETS, these findings highlight the importance of these enzymes and DNA methylation dynamics during neuronal development. Studies specifically focusing on these enzymes in postmitotic neurons have also highlighted the importance of DNA methylation in neuronal maturation and neuroplasticity, as discussed below.

## 4. DNA Methylation in Neuronal Maturation and Neuroplasticity

Since DNMTs are usually expressed in frequently dividing cells for the establishment and maintenance of DNA methylation patterns, it was originally perplexing to find considerable levels of maintenance enzyme *Dnmt1* and *de novo* enzyme *Dnmt3a* in adult postmitotic neurons [17,48]. However, several lines of evidence now support that these enzymes are essential for neural plasticity in postmitotic neurons.

Learning and memory are associated with alterations in the methylation status of various plasticity-related genes. Fear conditioning causes the memory suppressor gene *Pp1* to become rapidly methylated, resulting in the repression of this gene. This response is dependent upon DNMT activity given that chemical inhibition of this family of enzymes abolishes this effect [49]. Another plasticity-related gene whose methylation status is affected in response to contextual fear conditioning is *Bdnf*, which undergoes promoter-specific methylation that corresponds with isoform-specific changes in gene expression [50,51]. This regulation of isoform-specific expression of *Bdnf* is DNMT-dependent since chemical inhibition of DNMTs results in alterations in promoter methylation and isoform expression [51]. The importance of DNMTs in eliciting the changes in gene expression necessary for long-term memory formation is corroborated by the observed increase in *Dnmt3a* and *Dnmt3b* expression in the hippocampus in response to fear conditioning in both mice and rats [49,52], and inhibiting DNMT activity, via intrahippocampal infusion of a DNMT inhibitor, abolishes memory formation [49]. 

Double conditional knockout of *Dnmt1* and *Dnmt3a* in 2-3 week postnatal forebrain excitatory neurons results in a decrease in DNA methylation, the misexpression of genes, a reduction in soma size, alterations in synaptic function, and deficits in learning and memory [53]. Although *Dnmt1* single conditional knockout in 2-3 week postnatal forebrain excitatory neurons does not appear to affect learning and memory nor a variety of cellular and molecular characteristics in the hippocampus or cortex, including methylation levels, neuronal survival, gene expression, soma size, or synaptic function (LTP and LTD) [52,53,54], this loss of *Dnmt1* does result in an anxiolytic and antidepressant-like phenotype, suggesting that cell types, brain regions, or neuronal circuits that underlie specific behaviors can be differentially vulnerable to the loss of certain DNMTs [55]. A consensus on the consequence of *Dnmt3a* loss in postmitotic forebrain excitatory neurons remains to be determined, as one group reports no changes in any parameters assessed (DNA methylation, gene expression, soma size, synaptic function, and learning and memory), while another group finds deficits in LTP in conjunction with associative and episodic memory dysfunction [52,53].

The importance of TETs in postmitotic neurons has also been ascertained. TET1 is thought to be involved in neuronal activity-induced DNA demethylation and gene expression changes since shRNA-mediated knockdown of endogenous *Tet1* in the dentate gyrus (DG) abolishes electroconvulsive stimulation (ECS)-induced demethylation of a *Bdnf* promoter [56]. These findings are in agreement with another study that has shown that *Tet1* knockdown in hippocampal neurons leads to the hypermethylation of a *Bdnf* promoter and subsequent repression of transcription from this promoter [57]. Studies on TET3 function have confirmed that this TET family member, which is the most highly expressed TET in the brain, is essential in mediating neuronal activity-dependent gene expression programs. When mice undergo extinction training, there is a significant increase in *Tet3* mRNA in the cortex, and when *Tet3* is knocked down via lentiviral plasmids in the infralimbic prefrontal cortex (ILPFC), mice display impaired fear memory extinction. Furthermore, inhibiting NMDAR activity blocks the increase in *Tet3* mRNA levels during fear memory extinction, suggesting that the rise in *Tet3* expression occurs via an activity-dependent, NMDAR-mediated pathway. Notably, fear acquisition or fear extinction alters 5hmC levels at genomic locations that contain CA or CT dinucleotides more so than regions that contain CG dinucleotides [58]. *Tet3* expression levels correlate with neuronal activity as well; an increase in synaptic transmission correlates with an increase in *Tet3*, but not *Tet1* or *Tet2*, mRNA and protein levels. When *Tet3* is knocked down from hippocampal neurons in culture, miniature excitatory postsynaptic current (mEPSC) amplitudes are significantly larger than controls, and the reciprocal effect occurs when *Tet3* is overexpressed. *Tet3* is also essential for the maintenance of homeostatic synaptic plasticity; when *Tet3* is knocked down in hippocampal cultured neurons, promoter IV of *Bdnf* is hypermethylated, resulting in a decrease in expression from this promoter [57]. And finally, the last TET family member, TET2, is thought to play a role in the hydroxymethylation of developmentally regulated genomic loci. With the use of *Tet2* knockout mice, it was found that this TET family member is responsible for the oxidation of a large fraction (~20%) of CG genomic regions that gain hydroxymethylation status over development and aging [13]. Taken together, these findings implicate that essential, locus-specific alterations in DNA methylation during neuronal plasticity and maturation are mediated by DNMTs and TETs.

## 5. Deciphering DNA Methylation: An Introduction to Methyl-CpG Binding Domain Proteins

Methylation is thought to influence transcription by altering DNA-protein interactions, such as the binding of transcription factors [59] or the recruitment of proteins that bind methylated DNA [60,61,62,63]. The families of proteins that bind methylated DNA to mediate the molecular consequences of this epigenetic mark, canonically known as “readers” of DNA methylation, are the SET- and Ring finger-associated (SRA) domain protein family, the Kaiso protein family, and the methyl-CpG binding domain (MBD) protein family [60,64]. MBD proteins, consisting of seven proteins (MBDs 1–6) and methyl-CpG-binding protein 2 (MeCP2), were originally demarcated as a family due to their shared, highly conserved MBD domain [60,65]; however, it has since been discovered that two of the family members, MBD5 and MBD6, do not bind to methylated DNA [66] and that some of these family members also have the ability to bind to other forms of DNA methylation in addition to 5mCG through their MBD: a point mutation in the MBD of MBD3 renders it able to bind to unmodified cytosine, 5mCG, and 5hmCG [67,68]; and MeCP2 has recently been demonstrated to bind with high affinity to 5mCH and 5hmCH [29,37,69,70]. In this review, we will focus on the most well-studied reader of DNA methylation, MeCP2, given its recognized importance in neuronal function and unique ability to bind to a neuronally-enriched form of DNA methylation, 5mCH.

## 6. MeCP2’s Role in Neuronal Function

MeCP2 is a nuclear protein found in various tissues in mammals [71,72], with highest expression in the brain where it is half as abundant as nucleosomes [73]. Within the brain, MeCP2 protein levels are seven times higher in neurons than glia, underscoring the importance of this protein in neuronal function [72]. However, various studies have shown that MeCP2 is also important for glial function as well [74,75,76,77,78]. The levels of MeCP2 in the mammalian brain increase over postnatal development, suggesting that MeCP2 is important for synapse maturation [72,79]. Clear evidence for the importance of this gene in the CNS came with the discovery that mutations in *MECP2* cause Rett Syndrome (RTT), a severe neurological disorder that affects 1 in 10,000 live female births, making it one of the most common causes of intellectual disability in females [80]. Given that *MECP2* is an X-linked gene, RTT is almost exclusive found in females, as hemizygous loss of MeCP2 function in males leads to severe neonatal encephalopathy and death [80,81,82,83,84].

RTT is characterized by normal development for the first 6 months of life followed by regression and symptomatic presentation, which includes loss of previously acquired purposeful hand skills and spoken language, gait abnormalities, and hand stereotypies [85,86]. Postnatal deceleration of head growth is another clinical feature of RTT [85,86], and radiological and pathological examinations have determined that this is due to a reduction in brain size [87]. Given the absence of degenerative features, this change in brain volume is attributed to a reduction in the number and length of dendrites, resulting in more densely packed neurons [87,88]. These changes are brain region- and cortical layer-specific, as various groups have observed changes in only a subset of areas examined [87,89]. The deficits observed in cortical layers III and V are indicative of deficits initiating during the second period of cortical development when lower layers are refining their connectivity with layer III postnatally. Additionally, these dendritic alterations do not worsen over adulthood, indicating that RTT is not a progressive disease after development [87]. Supporting a functional consequence of these anatomical deficits are electrophysiological studies in neurons derived from RTT induced pluripotent stem cells (iPSCs) that have found a decrease in the amplitude and frequency of spontaneous excitatory transmission [90]. Taken together, these findings in RTT patients are clear indications of a disrupted neuronal network and suggest that RTT is a neuronal maturation and/or maintenance disorder.

To understand the molecular underpinnings of RTT, mice lacking *Mecp2* or carrying RTT-associated mutations in *Mecp2* have been developed, and the majority of clinical features are observed in these whole-body *Mecp2*-mutant mice, such as normal development during early life followed by symptomatic presentation of altered gait, motor incoordination, hindlimb clasping, and cognitive deficits [91,92,93]. Loss of *Mecp2* from only the CNS either embryonically (embryonic day 12, E12) or postnatally (postnatal day 21, P21) results in phenotypes that are indistinguishable from the loss of *Mecp2* from the whole-body [91], emphasizing the importance of this MBD protein in neuronal function and maintenance. In addition to behavioral phenotypes, *Mecp2*-mutant mice also display pathological and histological features found in RTT patients, such as a smaller brain size [91,92,93] and a reduction in the number and length of dendrites [94,95]. Furthermore, the dendritic spines of *Mecp2*-null mice are reduced in number and altered in morphology, resembling immature spines [94,95,96,97]. These alterations in dendritic branches and dendritic spines are suggestive of neuronal connectivity deficits in *Mecp2*-null mice, and in fact, electrophysiological experiments corroborate this. In *Mecp2*-null mice, a reduction in excitatory synapses, as well as the quantal excitatory transmission at those synapses, contributes to a reduction in the spontaneous firing rate of cortical neurons [93,98,99,100]. In addition to synaptic dysfunction, there are also deficits in LTP and LTD, which is thought to occur subsequently to the synaptic dysfunction [99]. The alterations in LTP and LTD in RTT harken back to LTP and LTD deficits observed in DNMT and TET loss of function mice. Given these neuronal deficits, the molecular perturbations that occur with *MECP2* mutations or loss have been intensely studied in order to understand the biological function of this protein, as will be discussed below.

## 7. Proposed Molecular Functions of MeCP2

### 7.1. Multiple Functions have been proposed for MeCP2

This year marks the 25th anniversary of the discovery of MeCP2 [101]. Over this span of time, ~1000 papers have been published with MeCP2 in the title. This large body of research has greatly advanced our understanding of MeCP2; however, a consensus on the specific molecular function of MeCP2 has yet to be reached, with evidence supporting MeCP2’s role as a transcriptional repressor, transcriptional activator, chromatin organizer, regulator of alternative splicing, and miRNA processor [102]. In this review, we will focus on the three prevailing models: transcriptional repressor, transcriptional activator, and chromatin organizer.

### 7.2. MeCP2: A Repressor?

A screen for proteins that bind methylated DNA led to the discovery of MeCP2, given its high affinity for DNA methylation, as it is capable of binding to DNA with a symmetrically methylated 5mCG [101] through its 85kDa MBD [64]. In this initial work, it was also found that the nuclear localization of MeCP2 mirrored that of 5mCG, given its high concentration at pericentromeric heterochromatin, which is known to contain ~40% of all genomic 5mCG at major satellite DNA [101]. Another functional domain of MeCP2 is the transcriptional repression domain (TRD), which has been shown to exhibit long-range repression, up to 2kb away from a transcriptional start site [103]. A small portion of the TRD interacts with the histone deacetylase (HDAC)-containing nuclear receptor co-repressor (NCOR) and silencing mediator of retinoic acid and thyroid hormone receptor (SMRT), which is termed the NCOR-SMRT interaction domain (NID) [104,105,106]. MeCP2 has also been shown to interact with the HDAC-containing repressor complex Sin3a; however, this binding site on MeCP2 remains to be determined [107,108]. The importance of the MBD and NID in MeCP2 function is supported by human genetics, since RTT-causing missense mutations, but not neutral mutations, predominantly cluster in these two domains [102,104]. Notably, each of the four RTT-causing missense mutations within the NID abrogate MeCP2’s ability to bind to NCOR-SMRT [104], highlighting the importance of this interaction for MeCP2 function. Since deacetylation of histone tails leads to transcriptional repression [109], these findings suggest a model in which MeCP2 acts as a molecular bridge to connect DNA methylation with chromatin changes that elicit repression [107].

Various studies support the idea that MeCP2 binds globally across the genome at methylated cytosine sites [37,69,73,110]. Chromatin immunoprecipitation (ChIP) followed by sequencing (ChIP-seq) has demonstrated that MeCP2 binding profiles scale with CG methylation density [69,73,111], with high levels of binding at CG methylation dense regions and lower levels of binding at unmethylated CG islands (CGIs) [73]. It has recently been discovered that, in addition to 5mCG, MeCP2 can also bind to 5mCH, specifically 5mCA, with high affinity [29,30,37,69,70]. When the genome-wide MeCP2 binding profiles in mouse cortical tissue were compared to base-pair resolution profiles of 5mC and 5hmC, it was found that MeCP2 binding globally correlates with 5mCG, 5mCA, and 5hmCA density, but not 5hmCG density. Additionally, misregulated genes in *Mecp2* knockout mice are enriched for MeCP2 binding and 5mCA in the wild type setting [29,30,37,69,70]. However, it remains to be determined which subsets of misregulated genes show this enrichment. One group finds that genes enriched with MeCP2 binding in the wild type setting tend to be long and also enriched with 5mCA, and that in the absence of *Mecp2* in cortical tissue, upregulated genes tend to be long, implicating MeCP2 as a transcriptional repressor [37]. An independent group confirms this finding in a different brain region, the hypothalamus, with the finding that MeCP2 binding in the wild type setting is enriched at long genes with high levels of 5mCA, and in *Mecp2* knockout mice, this gene group is upregulated [70]. However, another group reported that both upregulated and downregulated genes in *Mecp2*-null hypothalamus tissue are enriched for MeCP2 binding and 5mCH in the wild type setting, suggesting a more complex model in which MeCP2 acts as both an activator and repressor [69]. An important difference between the analyses carried out by these studies is that the former two groups analyzed MeCP2 and 5mCH enrichment within the gene bodies and surrounding regions of misregulated genes [37,70], whereas the latter group normalized intragenic MeCP2 and 5mCH enrichment to the surrounding regions of misregulated genes [69]. Given the fact that the raw data used by two groups with opposing conclusions [69,70] were identical, it is important to keep in mind that data analysis methods can critically influence the interpretation of MeCP2 function since gene expression changes are subtle in RTT.

### 7.3. MeCP2: An Activator?

In direct opposition of the repressor model, MeCP2 has also been proposed to be a transcriptional activator. In mouse neurons differentiated from *Mecp2*-null ESCs, there is a significant reduction of overall RNA synthesis, which was detected with the use of a radioactive ribonucleotide incorporation assay [112]. Similarly, in human neurons derived from *MECP2*-null ESCs, per-cell total RNA levels are significantly reduced, and this phenotype gets worse as time passes [113]. These findings are supported by an independent group that found a significant reduction of Serine5 phosphorylated RNA polymerase (a proxy for transcriptional activity) in *Mecp2*-null mouse neurons in vivo using array tomography (AT) imaging [114]. Additionally, gene expression studies from various brain regions of *Mecp2*-null mice have found that more genes are repressed than activated, supporting MeCP2’s role as an activator [115,116,117]. Furthermore, genome-wide promoter analysis revealed that the majority of MeCP2-bound promoters are associated with actively expressed genes that are not highly methylated, indicating that MeCP2 binding isn’t solely correlated with repression [118]. Molecularly, there is evidence supporting the idea that MeCP2 mediates its activator role through its interaction with cyclic AMP-responsive element-binding protein 1 (CREB1) at genes that are activated, but not at those that are repressed [115]. In support of MeCP2 potentially acting as an activator, MeCP2 was found to bind to 5hmCH (more specifically, 5hmCA) in vitro with the use of the electrophoretic mobility shift assay (EMSA) [37]. However, whether 5hmCA is associated with gene activation, like 5hmCG, or whether the binding of MeCP2 to 5hmCA is biologically significant, given the low percentage of 5hmC that is in the CH context, remains to be determined. Notably, multiple studies support the idea that MeCP2 does not bind to 5hmCG, either in vitro, with the use of EMSAs, fluorescent polarization, or mass spectrometry [37,67,68,119,120], or in vivo via correlating MeCP2 ChIP-seq profiles with base-pair resolution profiles of 5hmCG, which in fact shows an anticorrelation between the two [37]. Taken together with the previous section, these data indicate that more studies are necessary before classifying MeCP2 as a *bona fide* transcriptional activator or repressor.

### 7.4. MeCP2: A Transcriptional Mediator of Neuronal Activity?

In line with MeCP2 acting as a regulator of transcription are findings demonstrating MeCP2’s ability to regulate gene expression in an activity-dependent manner. In this model, at basal conditions, unmodified MeCP2 functions as a repressor when bound to gene regions. However, upon stimulation, post-translational modifications on MeCP2 lead to a reduction in MeCP2 binding and subsequent gene activation. This function of MeCP2 is particularly intriguing given the necessity of gene expression changes for the maintenance of long-lasting neuronal plasticity. One important neuronal plasticity-related gene affected by the loss of MeCP2 function is *Bdnf*, with transcript and protein levels being significantly reduced in the brains of *Mecp2*-knockout mice [69,115,121]. The levels of *Bdnf* appear to be critically important for the RTT phenotype, since increasing levels of *Bdnf* in *Mecp2*-null mice ameliorates electrophysiological dysfunction, improves locomotor deficits, and extends lifespan [121]. In the wild type setting, 5mCH levels and MeCP2 binding are enriched across the *Bdnf* locus, suggesting that MeCP2 binding to developmentally regulated 5mCH sites is critical for the regulation of genes that are important in the manifestation of RTT phenotypes [69]. Under basal conditions in neuronal cultures, MeCP2 is bound to the methylated promoter III of *Bdnf* where it is thought to act as a repressor of transcription through the recruitment of the Sin3a repressor complex, which is found to be present via ChIP [50,122,123]. However, in response to membrane depolarization, MeCP2 and the Sin3a complex disassociate from the *Bdnf* promoter, methylation levels decrease at the promoter, and *Bdnf* mRNA expression levels increase. Furthermore, the neurons subsequently display an increase in dendritic complexity and spine maturation, suggestive of an increase in neuroplasticity [50,122,123]. This release of MeCP2 from the *Bdnf* promoter is mediated by the phosphorylation of MeCP2 at serine 421 (pS421), given that the loss of this phosphorylation site on MeCP2 results in the loss of activity-dependent *Bdnf* mRNA expression [122,123]. This phosphorylated form of MeCP2 is brain-specific, as it is absent in a multitude of tissues, suggesting that this activity-dependent form of MeCP2 might be a contributing factor in why RTT is predominantly a neurological disorder.

In the mouse brain in response to neuronal activity, 10–30% of total MeCP2 becomes phosphorylated at S421 [110]. However, the genomic binding profile of MeCP2 remains virtually unchanged in response to neuronal activity in the mouse brain, in opposition to what was observed at *Bdnf*. To investigate the importance of MeCP2 phosphorylation at S421 in vivo, knock-in mice have been created in which S421 is converted to alanine (S421A), preventing the phosphorylation of this site. Cortical pyramidal neurons in these mutants have increased dendritic complexity and an increase in the amplitude of miniature inhibitory postsynaptic currents (mIPSCs), highlighting the importance of this phosphorylation site on MeCP2 in neuronal development and function. When MeCP2 binding is assessed in wild type and MeCP2-S421A primary cultures in response to neuronal activity, it was found that the binding patterns are maintained in the mutant, implying that this phosphorylation site is not necessary for the release of MeCP2 from bound sites in response to neuronal activation [110]. Additionally, the employment of a more sensitive and targeted approach, ChIP-qPCR of wild type MeCP2 and MeCP2-S421A at activity-dependent genes in neuronal cultures in response to stimulation, found that MeCP2 binding patterns are similar across the WT and mutated forms of MeCP2. This suggests that additional phosphorylation events might contribute to the release of MeCP2 from chromatin or that specific stimulation paradigms are required to elicit a release. Furthermore, activity-induced gene expression programs are not altered in the MeCP2-S241A mutant mice, suggesting that this single phosphorylation site is not responsible for the release of MeCP2 from genomic regions [110]. And in fact, three additional phosphorylation sites on MeCP2 have been described: S86, S247, and T308. Phosphorylation of these sites on MeCP2 are differentially induced in response to various forms of stimulation, such as robust neuronal activity, exogenous BDNF, and chemically induced elevations of cAMP, suggesting that these phosphorylation sites induce different expression patterns in response to different external stimuli. Supporting the importance of one of these phosphorylation sites is the finding that mice carrying a T308A mutation in MeCP2 display RTT phenotypes, constitutive NCOR-SMRT binding to MeCP2, and a reduction in the expression of activity-induced genes [124]. In addition to the S421, S86, S247, and T308 phosphorylation sites on MeCP2, additional phosphorylation sites and post-translational modifications on MeCP2 have been identified, suggesting that this protein is regulated in various ways [125,126]. Taken together, these findings highlight the potential importance of MeCP2 as a mediator of gene expression changes in response to neuronal activity; however, much remains to be explored to determine the transcriptional effects of the various post-translational modifications on MeCP2. 

### 7.5. MeCP2: An Architectural Protein?

In addition to the MBD, MeCP2 has three additional domains that enable it to bind to DNA. These are three basic clusters containing AT-hook-like domains, which bind to AT-rich sequences of DNA [101,127]. The recombinant form of one of these AT-Hook domains, AT-Hook 2 (amino acids 265–272), has been shown to independently bind to DNA, and a RTT-associated mutation in this domain (R270X) abolishes this binding ability. This also relates to in vitro work that has demonstrated the necessity of A/T rich DNA (four or more A/T bases) adjacent to a methylated CpG site for efficient MeCP2 binding [128]. Another region of MeCP2 implicated in chromatin organization is the C-terminal portion of the MBD that has been found to bind to ATRX, a chromatin remodeler that is mutated in α-thalassemia/mental retardation, X-linked syndrome (ATRX syndrome) [129]. MeCP2 is thought to be important for the localization of ATRX to pericentric heterochromatin, as loss of MeCP2 function in *Mecp2*-null mice or in mice carrying several RTT-associated point mutations in *Mecp2* abolishes ATRX localization to pericentric heterochromatin in the brain [127,129]. 

Recombinant MeCP2 binds, compacts, and oligomerizes nucleosomal arrays in vitro [130,131,132]. Compaction by MeCP2 comes in two forms, the clustering of nucleosomes and the formation of DNA-MeCP2-DNA complexes [131]. One striking feature of nucleosomal array compaction by MeCP2 is the formation of chromatin loops (free DNA emanating from clusters of nucleosomes) [132,133], which are implicated in the silencing of loci in vivo [133]. Further supporting MeCP2’s role in chromatin compaction is the finding that ectopic expression of the MBD of MeCP2 in mouse myoblasts is sufficient to cause the clustering of pericentric heterochromatin [134]. In a comprehensive study evaluating the effect of 21 RTT-associated mutations on MeCP2’s ability to bind and cluster chromocenters in mouse myoblasts, it was found that half of the mutations led to deficits in chromatin binding and two-thirds led to a decrease in the ability to cluster chromocenters [135]. When the nuclear localization of heterochromatin protein 1 (HP1), a protein essential for heterochromatin packaging, and MeCP2 were compared over cellular differentiation using a culture system of myogenesis, it was found that both HP1 (specifically the γ isoform (HP1γ) and MeCP2 redistributed to heterochromatic foci around the same time [136]. Additionally, MeCP2 interacts through its N-terminal domain (the first 55 amino acids) to the chromo shadow domain of HP1 [136]. Taken together, these studies suggest that MeCP2 plays a role in the organization and compaction of nucleosomes, especially in heterochromatin, and suggest that loss of function of MeCP2 would lead to decondensation of heterochromatin. 

However, although few in number, studies examining the effects of MeCP2 loss on heterochromatin compaction in mouse neurons have found an increase, rather than decrease, in heterochromatinization. Using array tomography (AT) imaging in mosaic RTT mouse females, there was found to be an increase of DAPI density in heterochromatin in *Mecp2-*null CA1 pyramidal neurons of the hippocampus, which is indicative of an increase in condensation of heterochromatin. Additionally, it was found that heterochromatin volume and nuclear volume are negatively correlated, suggesting that chromatin condensation reduces nuclear size. AT imaging of *Mecp2*-null CA1 pyramidal neurons of the hippocampus also detected a redistribution of trimethylation of histone H4 at lysine 20 (H4K20me3) to pericentromeric heterochromatin, which is a region occupied by MeCP2 in the wild type setting. This redistribution of H4K20me3 in *Mecp2*-null neurons is dramatic, with 65% of total H4K20me3 changing its localization to dense heterochromatin, while there was a negligible increase (11%) in overall H4K20me3 nuclear levels. Additionally, there is a significant redistribution of trimethylation of histone H3 at lysine 9 (H3K9me3) to pericentromeric heterochromatin, as well as a slight increase (10%) in total H3K9me3 abundance [114]. This change in nuclear organization is cell type-specific, given that granule cells of the dentate gyrus show a similar pattern that was observed in CA1 pyramidal neurons, but this alteration is absent in granule cells of the cerebellum. Another study found that although heterochromatin size and numbers are affected embryonically and perinatally in *Mecp2*-null neurons, these effects are not found later during development. The importance of chromocenter size is highlighted by a study that found an increase in heterochromatin size after induced depolarization of primary neuronal cultures; however, this effect is absent in *Mecp2*-deficient primary neurons [137]. Given that the findings from the few studies on heterochromatic changes in neurons are disparate, more research is warranted to determine the extent of organizational defects in RTT.

Further support for MeCP2’s role in chromatin organization comes from studies that have compared MeCP2 to histone H1 (H1), an essential component of chromatin that binds to linker DNA and organizes nucleosomes into higher order structures. H1 is present at the level of one molecule per nucleosome in most somatic cells, except for neurons, where it is present at the level of one molecule for every two nucleosomes [73,138,139]. in vitro, MeCP2 is able to displace H1 from preassembled chromatin [103,132,140], but not vice versa [132], suggesting that H1 and MeCP2 compete for binding to linker DNA regions. Additional in vitro support for this comes from studies using nucleosomal arrays in which human MeCP2 is found to bind to 11bp of linker DNA at the entry-exit site, protecting this portion of linker DNA from micrococcal nuclease (MNase) digestion, and a RTT-associated mutation in the MBD, MeCP2-R106W, results in a loss of protection of the linker DNA from MNase digestion [141]. Furthermore, linker DNA is essential for MeCP2’s ability to properly bind to nucleosomes, and this binding induces a variety of conformational changes to linker DNA [141]. Additionally, of all the canonical histone components, MeCP2 is in closest proximity to H3, whose N-terminal region is in closest proximity to the linker region [141]. When mouse fibroblasts stably expressing fluorescent MeCP2 are challenged with recombinant H1, and vice versa (mouse fibroblasts stably expressing fluorescent H1 are challenged by recombinant MeCP2), it was found that MeCP2 and H1 compete for binding sites, but that, similar to in vitro findings, MeCP2 is more effective at expelling H1 than the reverse [132]. Notably, in mouse fibroblasts, H1 and MeCP2 colocalize at pericentromeric heterochromatin [132]. Furthermore, MeCP2 induces nucleosomal array compaction in a zigzag-folding pattern that is very similar to the manner in which H1 induces compaction, which is thought to be essential for 30nm fiber formation that is the basic building block of heterochromatin [132]. Additionally, in *Mecp2* knockout neurons from the cortex, H1 protein levels double, reaching the abundance of 1 molecule per nucleosome [73]. H1 levels do not change in unsorted nuclei from the cortex, which contains both glia and neurons, suggesting that this change is specific to neurons [73]. This suggests that the lower levels of H1 in neurons are due to the presence of MeCP2, and that in the absence of MeCP2, sites are now open for histone H1 occupancy [73]. However, this notion remains to be fully addressed in vivo in the CNS.

### 7.6. MeCP2: A Multifunctional Protein

Twenty-five years of research on MeCP2 has led to many interesting insights into the complex, multifaceted function of this protein, generating numerous hypothesized molecular models. And yet another model is emerging—a synergistic model that treats many of the proposed functions as an integrative piece of MeCP2’s function, instead of treating them as mutually exclusive entities. It is possible that MeCP2 is predominantly an architectural protein that is important for regulating chromatin structural changes that aid in the increase or decrease of gene expression necessary for long-lasting plasticity in response to neuronal activity. Therefore, in this model, the transcriptional activator or repressor effects that have been attributed to MeCP2 would be secondary to its organizational role, similar to the function of CCCTC-binding factor (CTCF) [32,142]. However, to prove such a model, additional studies are necessary to interrogate the relationship between chromatin architectural changes and alterations in gene expression in a homogeneous population of neurons, or even at single-cell resolution, in the mammalian brain in RTT.

## 8. Conclusions

Neurons are unique in that they readily modify their gene expression programs to enable long-lasting plasticity throughout their lifetime. A key contributing factor to this characteristic is a neuron’s unique, adaptable methylome. Two families of enzymes that regulate DNA methylation status, DNMTs and TETs, are essential for normal neuronal function. Additionally, a neuronally enriched “reader” of DNA methylation, MeCP2, plays a particularly important role in mediating the biological functions of the brain’s distinctive methylome. 

## Figures and Tables

**Table 1:** Broad methylation features in the adult mouse brain.

Methylation Type and Context	Percentage of All Cytosines in this Context	Percentage of Methylated Cytosines in this Context	Generally Associated with Repression or Activation	Relative Enrichment (↑) or Depletion (↓) at Each Broad Genomic Feature				
				Enhancers	Promoters	Actively Expressed Gene Bodies	Silenced Genes	Repetitive DNA
5mCG	~2.9%	~62%	Repression	↓	↓	↓	↑	↑
5mCH	~1.3%	~25–40%	Repression	↓	↓	↓	↑	↑
5hmCG	~0.9%	~10–20%	Activation	↑	↓	↑	↓	↓
